# Naturally Isolated Sesquiterpene Lactone and Hydroxyanthraquinone Induce Apoptosis in Oral Squamous Cell Carcinoma Cell Line

**DOI:** 10.3390/cancers15020557

**Published:** 2023-01-16

**Authors:** Afshan Shams, Ayaz Ahmed, Ajmal Khan, Shariqa Khawaja, Najeeb Ur Rehman, Asma Saleem Qazi, Adnan Khan, Sami Bawazeer, Syed Abid Ali, Ahmed Al-Harrasi

**Affiliations:** 1 Dr. Panjwani Center for Molecular Medicine and Drug Research, International Center for Chemical and Biological Sciences, University of Karachi, Karachi 75270, Pakistan; 2 Natural and Medical Sciences Research Center, University of Nizwa, Birkat-Al-Mouz, P.O. Box 33, Nizwa 616, Oman; 3 Department of Biological Sciences, National University of Medical Sciences, Rawalpindi 46000, Pakistan; 4 Department of Microbiology, University of Karachi, Karachi 75270, Pakistan; 5 Department of Pharmacognosy, Faculty of Pharmacy, Umm Al-Qura University, Makkah 21955, Saudi Arabia; 6 Third World Center for Science and Technology, H.E.J. Research Institute of Chemistry, University of Karachi, Karachi 75270, Pakistan

**Keywords:** oral squamous cell carcinoma, costunolide, aloe emodin, *Lycium shawii*, caspase, anti-apoptotic, pro-inflammatory genes

## Abstract

**Simple Summary:**

Globally, oral cancer is one of the leading causes of mortality, especially in Asian countries. Among the various types, oral squamous cell carcinoma (OSCC) is more prevalent. The available treatment regime available to tackle OSCC has side effects and resistance issues. This requires a study for an alternative pharmacophore as a potential anticancer compound with the least amount of toxicity. So, in this study, two natural compounds, costunolide (CE) and aloe emodin (AE), were screened for their anticancer potential against OSCC (CAL 27) cells. Both compounds, CE (32 µM) and AE (38 µM), inhibited the cancer cell lines at low doses, as comparable to fluorouracil (97.76 µM), and were found to be non-toxic on normal cells. The active compounds induced apoptosis, as well as morphological and nuclear changes in the cancer cells. At the gene level, these compounds enhance the expression of the apoptotic genes and reduces the expression of anti-apoptotic and metastatic genes. Thus, these compounds might have the potential to become promising anticancer candidates.

**Abstract:**

Oral squamous cell carcinoma (OSCC) is one of the most prevalent cancers worldwide, especially in Asian countries. The emergence of its drug resistance and its side effects demands alternatives, to improve prognosis. Since the majority of cancer drugs are derived from natural sources, it provides a window to look for more biocompatible alternatives. In this study, two natural compounds, costunolide (CE) and aloe emodin (AE), were isolated from the stem of *Lycium shawii.* The compounds were examined for their anticancer and apoptotic potentials against OSCC (CAL 27) cells, using an in vitro analysis, such as a MTT assay, scratch assay, gene, and protein expressions. Both compounds, CE and AE, were found to be cytotoxic against the cancer cells with an IC_50_ value of 32 and 38 µM, respectively. Moreover, the compounds were found to be non-toxic against normal NIH-3T3 cells and comparable with the standard drug i.e., 5-fluorouracil (IC_50_ = 97.76 µM). These compounds were active against normal cells at higher concentrations. Nuclear staining displayed the presence of apoptosis-associated morphological changes, i.e., karyopyknosis and karyorrhexis in the treated cancer cells. Flow cytometry results further confirmed that these compounds induce apoptosis rather than necrosis, as the majority of the cells were found in the late apoptotic phase. Gene and protein expression analyses showed an increased expression of apoptotic genes, i.e., BAK, caspase 3, 6, and 9. Moreover, the compounds significantly downregulated the expression of the anti-apoptotic (BCL-2 L1), metastatic (MMP-2), and pro-inflammatory (COX-2) genes. Both compounds have shown promising anticancer, apoptotic, and anti-migratory activities against the OSCC cell line (i.e., CAL-27). However, further in vivo studies are required to explore these compounds as anticancer agents.

## 1. Introduction

Oral cancer is the sixth most prevalent cancer worldwide, especially in South Asian countries, i.e., Bangladesh, Pakistan, India, and Sri Lanka [1]. It is an aggressively growing tumor leading to malignant neoplastic changes arising from the lip, oral squamous epithelium, or minor salivary glands [2]. Depending upon its origin, there are multiple types of oral cancers. The most prevalent subtype is oral squamous cell carcinoma (OSCC), which originates in the mouth and pharynx’s squamous epithelial layer. OSCC contributes to almost 90% of all oral cancer cases [3]. The risk factors associated with OSCC are mostly avoidable, including tobacco in all forms, betel quid chewing, and alcohol consumption [4]. It is estimated that, the risk is three times higher in tobacco smokers than non-smokers, which is synergistically increased when combined with alcohol consumption [5]. The approaches used to cure oral cancer are surgery, radiotherapy, and chemotherapy. Depending upon the advancement of the disease, chemotherapy is an optional treatment given in combination with other approaches. It has been shown to improve the overall survival in advanced oral cancer patients [6]. Despite having treatment alternatives, more than 0.37 million new lip and oral cavity cancer cases were diagnosed in 2020, and approximately 177,757 mortalities were reported, globally [7]. Furthermore, side effects and the rapid acquisition of resistance against chemotherapeutic drugs are additional challenges that hinder treatment [8]. 

Since ancient times, plants have endowed us with tremendous types of natural compounds with a pharmacological importance. It has been reported that only 10% of the existing plant species have been studied for the compounds against various diseases, including cancer [9]. Most importantly, due to their complex structure, plant secondary metabolites have a broad spectrum of anticancer activities [10]. Secondary metabolites, such as terpenoids, phenolics, and alkaloids, are small organic molecules that are non-essential for plant growth, reproduction, photosynthesis, or other primary functions [11]. The compounds used in this study are two secondary metabolites, i.e., costunolide (CE) and aloe emodin (AE), isolated from the stem of *Lycium shawii*. *L. shawii* Roem and Schult (native plant of the Arabian Gulf region; Solanaceae) belong to the genus *Lycium* (Solanaceae family) and consist of 90 species that are already employed for hypotensive, anti-diabetic, antioxidant, anticancer, anti-inflammatory, and anti-plasmodial activities [11,12,13,14]. They are also used in traditional medicine, to treat constipation, backaches, stomachaches, and fever [15,16,17].

CE is a 15-carbon sesquiterpene lactone molecule with three isoprene units [18]. It has already been reported for antibacterial, antiviral, antiprotozoal, and anticancer activities [19]. The anticancer property of CE has been extensively studied over the past decade. It has been reported to induce the cell cycle arrest and apoptosis in many cancers, namely, human lung squamous cell carcinoma, breast cancer, osteosarcoma, esophageal cancer, ovarian cancer, hepatocellular carcinoma, and colon cancer [20,21,22,23,24,25,26,27]. AE is a hydroxyanthraquinone that belongs to the phenolic class. The compound has been reported for its cell cycle arrest, induction of apoptosis, and reducing the cell migration in various cancers, including human bladder cancer, colorectal cancer, cervical cancer, and gastric cancer [28,29]. However, their effects on CAL-27 cells are still not reported in detail for their anticancer activity. The present study is focused on deciphering the apoptosis inducing and anti-migratory potential of CE and AE in OSCC cells.

## 2. Materials and Methods

### 2.1. Plant Collection and Identification

The stem of *L. shawii* was purchased from a souq (May 2015) and identified by the plant taxonomist Mr. Saif Al-Hatmi (Oman Botanical Garden, Muscat (OBGM), Oman). The voucher specimens of *L. shawii* (No: BSHR-05/2015) were deposited in the herbarium of OBGM.

### 2.2. Fractionation and Isolation

The air-dried powdered resin (1 kg) of *L. shawii* was finely extracted with methanol (MeOH) at room temperature and evaporated under reduced pressure, to yield a crude MeOH extract, which was successively fractionated into *n*-hexane, dichloromethane (CH_2_Cl_2_), ethyl acetate (AcOEt), *n-*butanol (*n*-BuOH), and aqueous fractions. The *n-*hexane fraction was subjected to silica gel column chromatography (CC) and eluted with *n-*hexane, followed by increasing/*n-*hexane/AcOEt (10, 20, 30, and 100%), to give four fractions LSF_A-D_. Fraction HB (0.5 g) was further purified by CC using *n-*hexane/EtOAc (10:90 to 20:80) to provide CE (45 mg). Similarly, the AcOEt fraction (25 g) was loaded over CC and eluted with a solvent system of increasing polarity, viz., *n-*hexane/AcOEt, AcOEt, AcOEt/MeOH, and pure MeOH, to obtained eight fractions (E_A-H_). Fraction ED was further subjected to CC purification using the eluent system of *n-*hexane/AcOEt (10:90 to 30:70) and afforded AE (60 mg) [30].

### 2.3. Preparation of the Compounds

The isolated natural compounds, i.e., CE and AE, were prepared in 100% DMSO (Dimethyl sulfoxide) with a stock concentration of 20 mM and stored in a freezer at −20 °C.

### 2.4. Cell Culture

Human tongue squamous cell carcinoma cell line (CAL 27; ATCC# CRL-2095) and the mouse embryo fibroblast cell line (NIH 3T3; ATCC# CRL-1658) were grown in high glucose Dulbecco’s Modified Eagle’s Medium (DMEM) (GIBCO, Auckland, New Zealand). Both cell lines were grown and maintained in the growth medium containing sodium bicarbonate (1.5 mg/mL), sodium pyruvate (1 mM), penicillin and streptomycin solution (1%), and heat-inactivated fetal bovine serum (FBS) (10%) (Sigma, St. Louis, MO, USA) at 37 °C in a humidified 5% CO_2_ incubator. Both cell lines were trypsinized, reaching an 80–90% confluence using a trypsin-EDTA solution (2×) (Hyclone, Logan, UT, USA) prepared in phosphate-buffered saline (PBS). Both cell lines were purchased from ATCC and provided by Biobank of the Dr. Panjwani Center for Molecular Medicine and Drug Research (PCMD).

### 2.5. Cytotoxicity Assay

The cytotoxicity of CE and AE against CAL 27 and NIH 3T3 cells was determined using a MTT assay [31]. The cells (1.5 × 10^4^/well) were seeded in 96-well tissue culture plates and incubated for 24 h. The next day, the monolayers were treated with different concentrations (250, 125, 62, and 31.5 µM) of test compounds and further incubated for 48 h. Then, 5-fluorouracil was used as a positive control, and 1% DMSO was used as a solvent control. Following treatment, the spent media was removed, and the plates were incubated with 0.5 mg/mL of MTT dye (Biobasic, Markham, ON, Canada) for 4 h. Later, the insoluble formazan crystals were solubilized in DMSO, and the absorbance was recorded at 570 nm using a SpectraMax microplate reader (Molecular Devices, San Jose, CA, USA). The half-maximal inhibitory concentration (IC_50_) was calculated using the EZ-Fit enzyme kinetics software by Perrella Scientific.

### 2.6. Cellular Morphological Analysis by Phase Contrast Microscopy

The CAL 27 cells (3 × 10^5^/well) were seeded in a 6-well tissue culture plate and incubated overnight. The next day, the spent medium was removed and the cells were treated with IC_50_ and IC_60_ doses of CE (i.e., 32 and 39 µM, respectively) and AE (i.e., 38 and 49 µM, respectively) in serum-free media for 48 h. The untreated CAL 27 cells were used as the negative control. The images were taken after treatment, at 0, 24, and 48 h using an Eclipse TE2000-S microscope (Nikon Corporation, Tokyo, Japan) at a 100× total magnification.

### 2.7. Nuclear Staining by DAPI

The nuclear changes in the treated cells were observed using DAPI staining, as described earlier [31]. Briefly, 1 × 10^6^ cells/well were seeded on cover slips, to form a monolayer. The next day, the cells were treated with compounds for 48 h. The cells were washed and fixed with 3.7% formaldehyde and stained with DAPI (1 µg/mL) for 10 min at room temperature. The nuclear changes were observed at 0, 24, and 48 h using an Eclipse 90*i* microscope (Nikon Corporation, Tokyo, Japan) with a total magnification of 400× at the Microscope Imaging Facility of the Dr. Panjwani Center for Molecular Medicine and Drug Research (PCMD). 

### 2.8. Assessment of Apoptosis by the YO-PRO-1/PI Staining

A Vybrant Apoptosis Assay Kit (Invitrogen, Waltham, MA, USA) was used to evaluate the percentage of the apoptotic and necrotic cells using the manufacturer’s protocol. The CAL 27 cells (1 × 10^6^/well) were seeded and treated with compounds for 48 h. Following treatment, all of the cells (suspended and adherent) were collected and treated with 1 µL of YO-PRO and 1 µL of propidium iodide (PI) and incubated on ice for 30 min. The analysis was carried out on FACSCalibur™ ( BD, Franklin Lakes, NJ, USA), equipped with CellquestPro software (5.2, BD Biosciences, San Jose, CA, USA). A total of 10,000 events were acquired from each sample, to evaluate the population percentage of the cells in the early apoptotic, late apoptotic, and necrotic phases [32]. 

### 2.9. Scratch Assay 

The anti-migratory potential of the compounds against the CAL 27 cells was analyzed by a scratch assay, as described before [33]. Briefly, the cells (3 × 10^6^/well) were plated in a 6-well plate and incubated overnight, to form a uniform monolayer. The next day, a scratch was made in the middle of the well using a 200 µL pipette tip, washed, and treated with compounds for 48 h, as described above. The untreated cells were used as the negative control. Five random points were selected in each scratch, to measure the closure percentage of the area using ImageJ 1.51 k software (Wayne Rasband, National Institute of Health, Bethesda, MD, USA). Following treatment, the images were taken at 0, 24, and 48 h using a TE 2000-S phase contrast microscope (Nikon Corporation, Tokyo, Japan) at a 100× total magnification. 

### 2.10. Gene Expression

The gene expression of the apoptotic and metastatic genes was analyzed after treating the CAL 27 cells (1 × 10^6^ /well) with CE and AE. Following treatment, RNA was extracted using TRIzol™ Reagent (Invitrogen, Waltham, MA, USA), by following the standard protocol. The RNA concentration and purity were determined using a Nanodrop-ND-2000 spectrophotometer (Thermo Fisher Scientific, Waltham, MA, USA). The complementary DNA (cDNA) analysis was made using RevertAid First Strand cDNA Synthesis Kit (Thermo Fisher Scientific, Waltham, MA, USA). Later, a PCR was used for the differential gene expression analysis and compared with the untreated control. The PCR products were resolved by electrophoresis on 1% agarose gel, containing 0.5 µg/mL ethidium bromide. A FluorChem FC3 system (Protein Simple, San Jose, CA, USA) was used to visualize the DNA bands and their integrated density was calculated using ImageJ 1.51 k software.

### 2.11. Immunocytochemistry

The differential expression of the BCL-2 L1 and caspase 3 proteins were evaluated in the compound-treated CAL 27 cells (4 × 10^4^ cells/well) in an 8-well chamber slide. Following treatment, the cells were treated with antibodies against the target proteins, as described earlier in a study [31]. The slide was then observed under an Eclipse 90*i* microscope (Nikon Corporation, Tokyo, Japan) at a 200× total magnification. The mean single cell intensity was calculated using ImageJ 1.51 k software (Wayne Rasband, National Institutes of Health, Bethesda, MD, USA). 

### 2.12. Statistical Analysis

All of the experiments were reproduced at least three times, to calculate the mean ± SD. The mean, standard deviation, one-way ANOVA, and student’s *t*-test were applied using IBM SPSS Statistics 21 software (IBM, New York, NY, USA). The *p*-values of less than 0.05 were considered as significant.

## 3. Results

### 3.1. Costunolide

**Figure 1:**
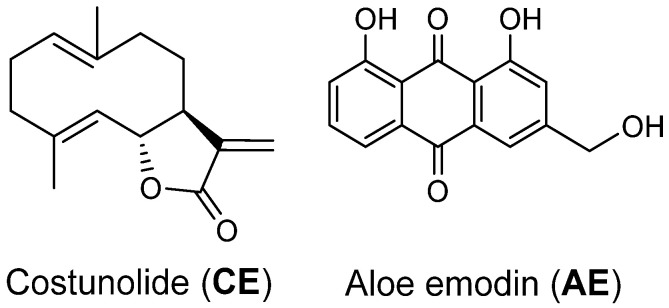
Structures of costunolide (CE) and aloe-emodin (AE).

The compound CE was isolated as a white amorphous powder from the hexane fraction of *L. shawii*. The ^1^H-NMR spectrum showed two proton peaks of the exo-methylene group at δ 6.24 (1H, d, J = 3.6 Hz, H-12), 5.50 (each 1H, d, J = 3.0 Hz, H-12), two olefinic protons at 4.84 (1H, dd, J = 11.0, 4.3 Hz, H-1), 4.72 (1H, d, J = 9.9 Hz, H-5), one oxygenated methine at 4.56 (1H, dd, J = 9.9, 8.7 Hz, H-6), and two methyl groups at δ 1.67 (3H, s, 15-CH3), 1.40 (3H, s, 14-CH3). The ^13^C-NMR spectrum displayed the presence of fifteen carbon signals, consisting of a carbonyl carbon signal at δ 170.4, six olefinic carbon signals at δ 141.4, 140.0, 136.9, 127.2, 126.0, and 119.6, four methylenes at 40.9, 39.4, 28.0, and 26.2, two methines at 81.9 and 50.4, and two methyls at 17.3 and 16.1. These spectral data suggested that CE should be a sesquiterpene compound bearing the exo-methylene lactone group. The ESI-MS (*m/z* 254.86 [M + Na]^+^) and ^1^H- and ^13^C-NMR spectral data of CE gave a molecular formula of C_15_H_20_O_2_. Based on these observations and the comparison of the data with those previously published [34,35], the structure of CE was identified as costunolide (Figure 1).

### 3.2. Aloe-Emodine

Compound AE (anthraquinone) was isolated as an orange-yellow solid from the ethyl acetate fraction of *L. shawii*. The molecular weight (292.85 [M + Na]^+^) was determined by ESI-MS, corresponding to a molecular formula of C_15_H_10_O_5_. The ^1^H-NMR spectrum showed five aromatic protons at 7.81 (1H, d, J = 1.6 Hz), 7.36 (1H, d, J = 1.6 Hz), 7.86 (1H, dd, J = 8.0, 1.2 Hz), 7.71 (1H, t, J = 8.0 Hz), and 7.32 (1H, dd, J = 8.0, 1.2 Hz), and two methylene protons at 4.84, (2H, d, J = 5.2 Hz). The ^13^C-NMR spectrum of AE determined the presence of fifteen carbon signals, consisting of a two carbonyl carbon signals at δ 192.6 and 181.7, five aromatic carbon signals at 137.7, 124.7, 121.3, 120.1, and 117.7, six quaternary carbon signals at 163.0, 162.5, 151.5, 133.7, 133.6, and 114.9, and one exo-methylene at 64.0. The final assignment of the compound AE was achieved by 2D NMR experiments (HMQC and HMBC), from which the compound was confirmed as aloe-emodin (Figure 1) [36,37].

### 3.3. Cytotoxic Effect of CE and AE

**Figure 2:**
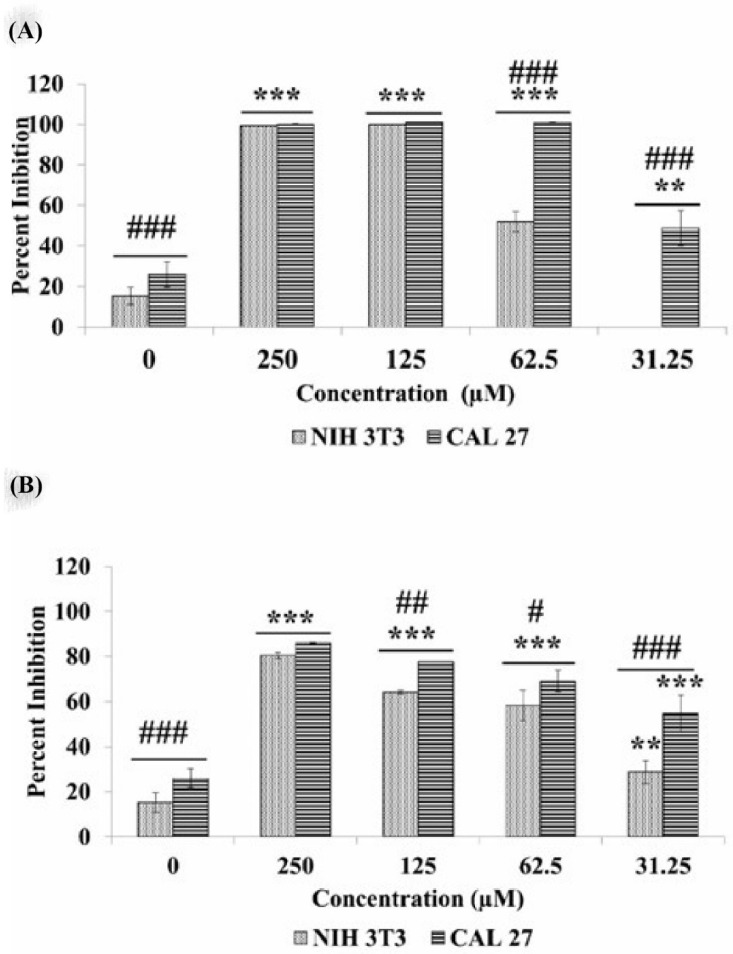
Inhibition percentage of the CAL 27 cells treated with different concentrations of CE (**A**) and AE (**B**). Each value is the mean ± SD of three experiments. ** *p* < 0.01, *** *p* < 0.001, in comparison with the untreated control CAL 27 cells. Whereas, ### *p* < 0.001, ## *p* < 0.01, # *p* < 0.05 comparing the two cell lines at the same dose. The short line above the bars shows same significance. If both the bars have same significance, we add a short line and put the symbol once.

The inhibition percentage of CAL 27 and NIH 3T3 cells by the test compounds were determined using a MTT assay and compared with the standard drug, i.e., 5-flurouracil. Compounds CE and AE were found active against the CAL 27 cells with an IC_50_ value of 32 and 38 µM, respectively, as compared to the standard drug (i.e., 97.7 µM; Table 1). The inhibition percentage of both compounds showed a dose dependent inhibition of the cancer cells, as shown in (Figure 2). However, their IC_50_ value on the normal cells, i.e., NIH 3T3, was found to be significantly high, as compared to the cancer cells (Table 1). Further experiments were conducted using IC_50_ and IC_60_ doses of CE (32 and 39 µM) and AE (38 and 49 µM), respectively.

### 3.4. CE and AE Induced Morphological Changes in the CAL 27 Cells

**Figure 3:**
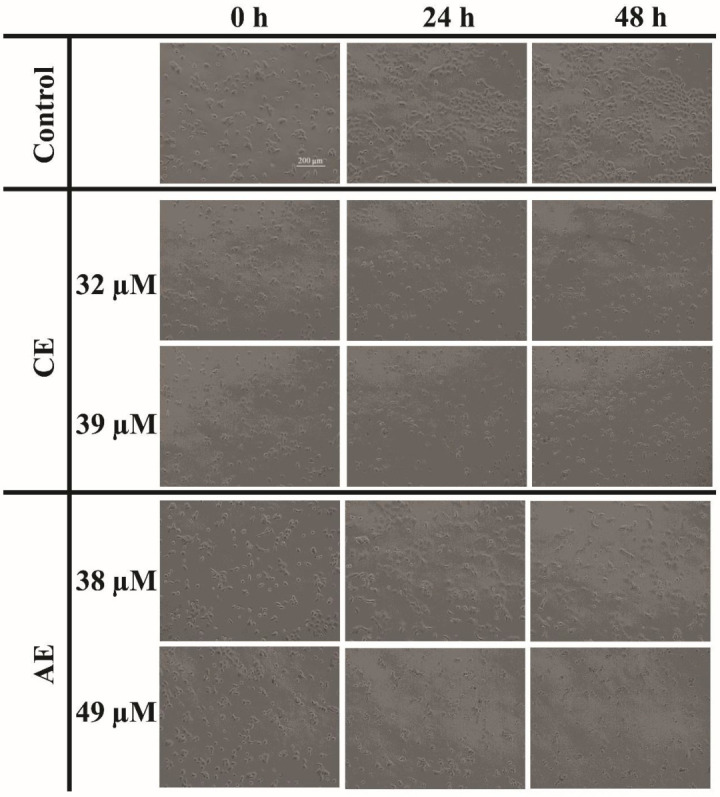
Phase contrast micrographs of the CAL 27 cells treated with IC_50_ and IC_60_ concentrations of the test compounds, in comparison with the untreated control cells. Scale bar: 200 μm.

**Figure 4:**
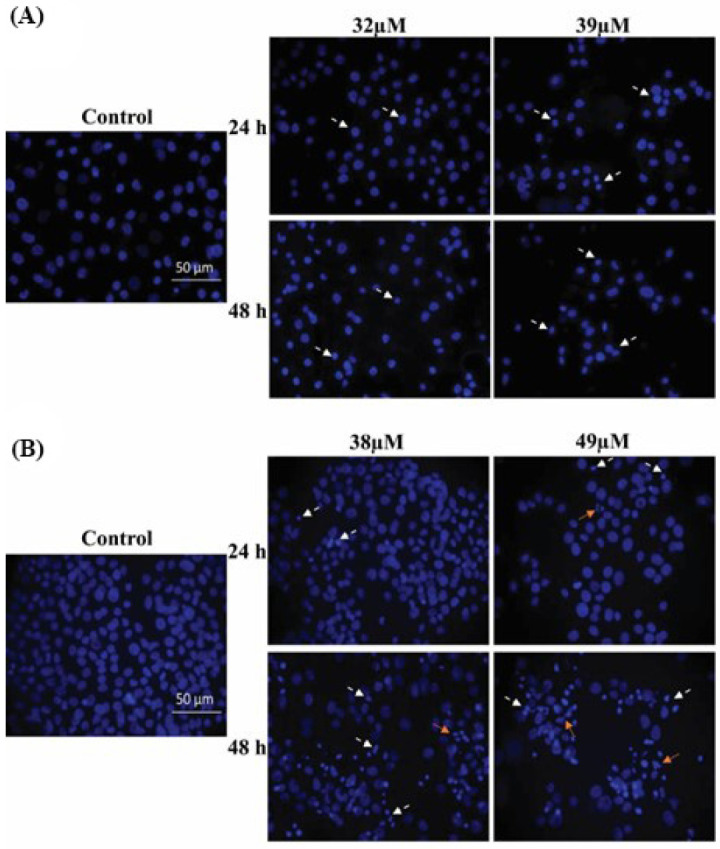
DAPI stained nuclei of the CAL 27 cells treated with IC_50_ and IC_60_ concentrations of CE (**A**) and AE, (**B**) in comparison with the untreated control. The dashed tail arrow (⤍) points to karyopyknosis and the dotted tail arrow (⤑) points to karyorrhexis. Scale bar: 50 μm.

The phase contrast micrographs show the well-adhered healthy untreated control cells. Following the CE and AE treatments at IC_50_ and IC_60_ doses, the CAL 27 cells were found to have shrunk, become detached, and were suspended in the medium (Figure 3). Moreover, karyopyknosis was observed in the DAPI-stained nuclei of the treated CAL 27 cells at both doses of each compound (Figure 4). Karyorrhexis was observed in the AE treated cells at both concentrations (Figure 4B). Whereas most of the untreated control nuclei were round and healthy with no karyopyknosis or karyorrhexis (Figure 4).

### 3.5. CE and AE Induce the Cell Death via Apoptosis

**Figure 5:**
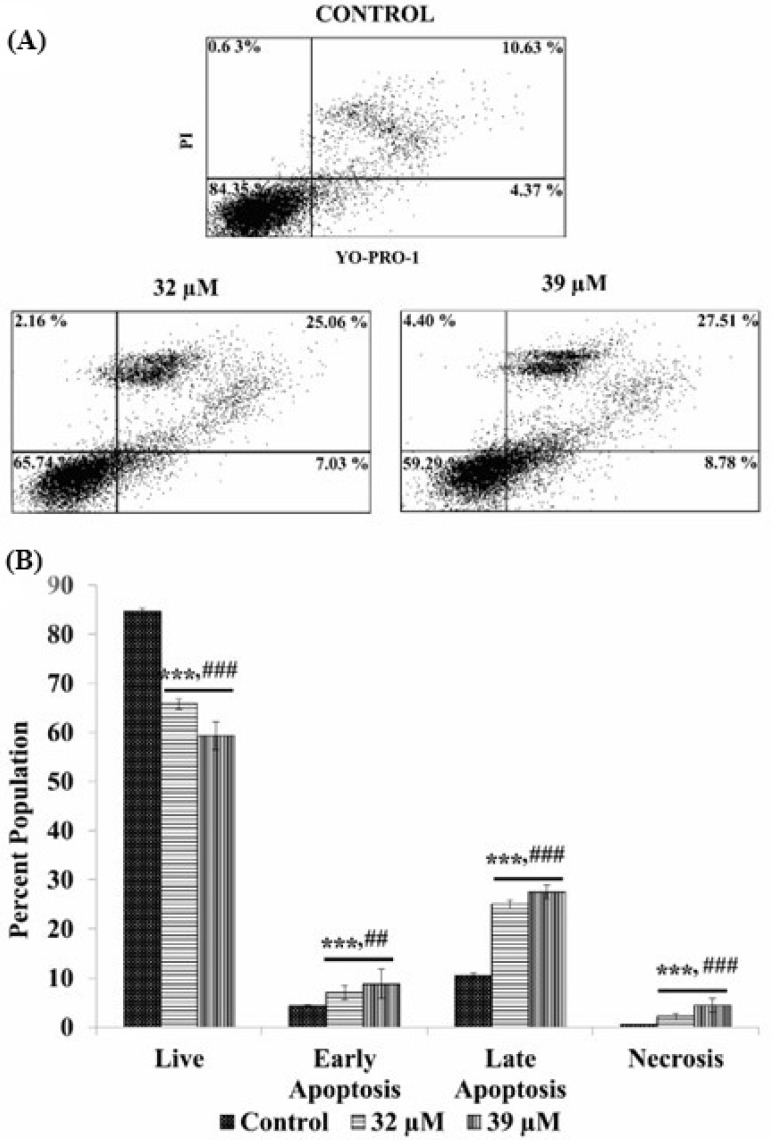
Apoptosis analysis in the CE treated CAL 27 cells by YO-PRO-1 and PI staining. Dot plots (A) showing the populations of cells in each quadrant. Population percentage of the live, early apoptotic, late apoptotic, and necrotic cells are represented graphically (**B**). Each value is the mean ± SD of three experiments. Where the statistically significant *p*-value was considered as: *** *p* < 0.001, as compared with the control and ### *p* < 0.001, ## *p* < 0.01 in comparison between the IC_50_ and IC_60_ doses of the compounds. The short line above the bars shows same significance. If both the bars have same significance, we add a short line and put the symbol once.

**Figure 6:**
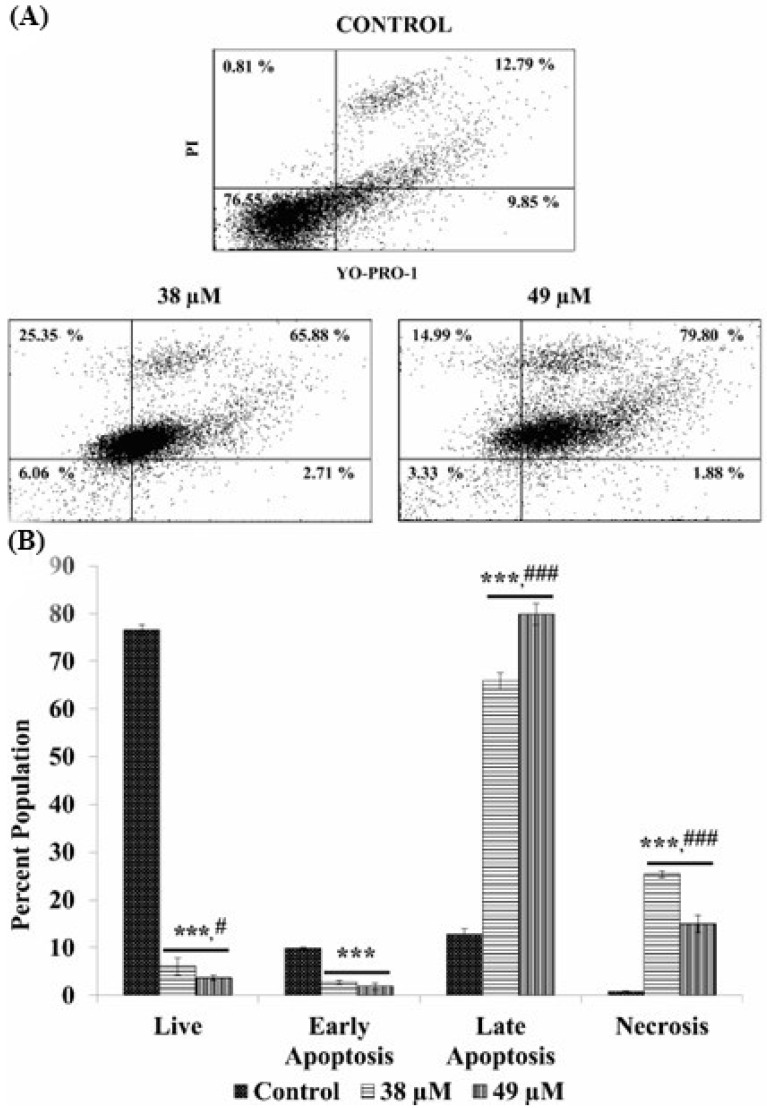
Apoptosis analysis in the AE treated CAL 27 cells by YO-PRO-1 and PI staining. Dot plots (A) showing the populations of cells in each quadrant. Population percentage of the live, early apoptotic, late apoptotic, and necrotic cells are represented graphically (**B**). Each value is the mean ± SD of three experiments. Where the statistically significant *p*-value was considered as: *** *p* < 0.001, as compared with the control and ### *p* < 0.001, # *p* < 0.05, in comparison between the IC_50_ and IC_60_ doses of the compounds. The short line above the bars shows same significance. If both the bars have same significance, we add a short line and put the symbol once.

The staining with YO-PRO and PI further confirmed the tendency of CE and AE to induce apoptosis in the CAL 27 cells. The flow cytometric analysis of the stained cells showed that a significant portion (*p* < 0.001) of the cellular population underwent apoptosis upon treatment with CE and AE, as compared to the untreated control cells (Figure 5A and Figure 6A). The percentages of the apoptotic cells were increased in the compound treated samples at different concentrations, compared with the control sample in a dose-dependent manner (Figure 5B and Figure 6B). Each dot plot consists of four quadrants representing the state of the cells, i.e., live (negative for both YO-PRO and PI; lower left), early apoptosis (YO-PRO positive and PI negative; lower right), late apoptosis (positive for both YO-PRO and PI; upper right), and necrosis (YO-PRO negative and PI positive; upper left). 

### 3.6. Gene Expression Analysis

**Figure 7:**
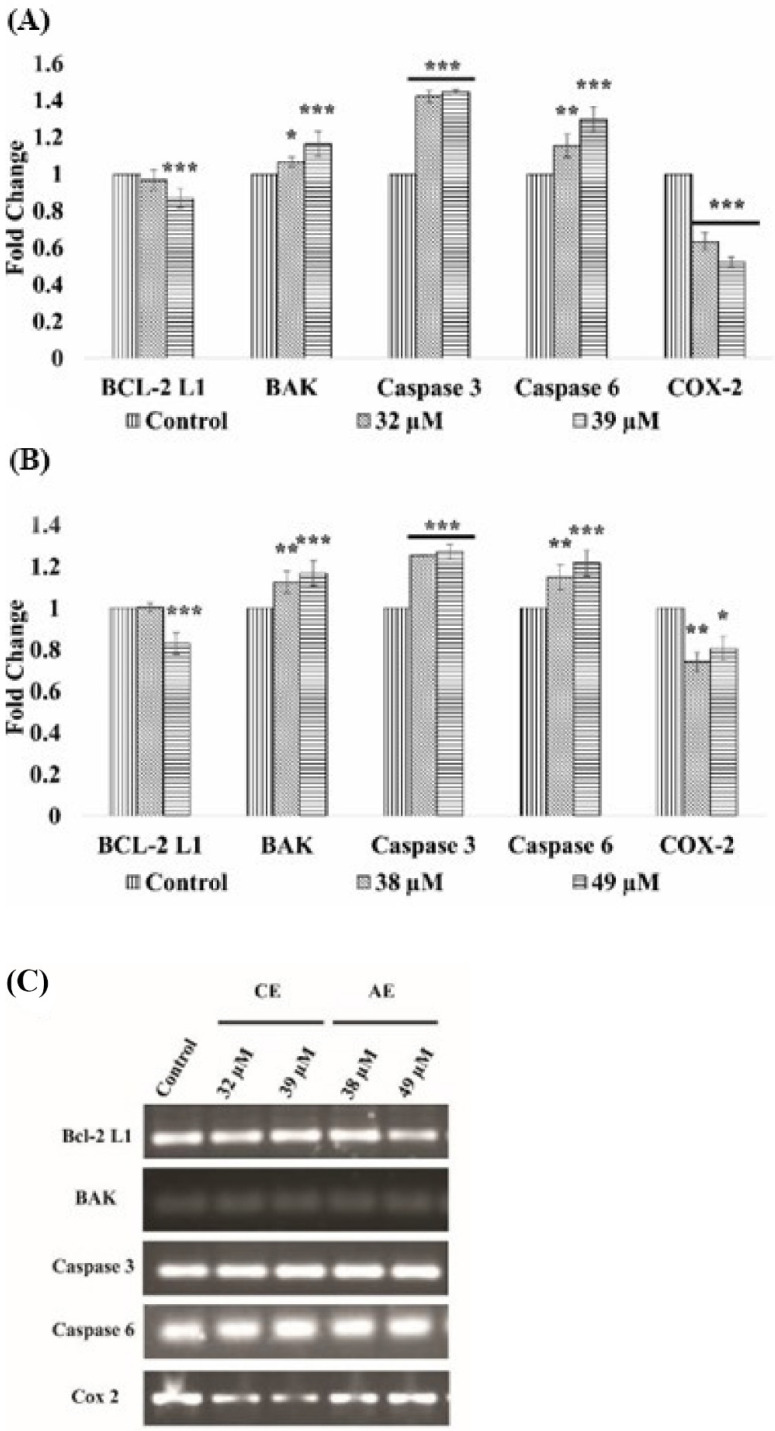
Difference in the gene expression of the CAL 27 cells after treatment with the compounds CE (**A**) and AE (**B**), in comparison with the control. Agarose gel images showing the band intensities (**C**). Each value is the mean ± SD of three experiments. Where the statistically significant *p*-value was considered as: *** *p* < 0.001, ** *p* < 0.01, and * *p* < 0.05, as compared with the control. The short line above the bars shows same significance. If both the bars have same significance, we add a short line and put the symbol once.

The mean integrated densities of the bands were used to calculate the fold change for each gene expression, compared with their respective control (Figure 7). A significant increase in the expression was observed in the set of apoptotic genes, i.e., BAK, caspase 3, 6, and 9. On the contrary, the expression of the pro-inflammatory (COX-2) had significantly decreased in a dose dependent manner in the compound treated cells. The anti-apoptotic (BCL-2 L1) gene expression was not significantly reduced at the IC_50_ concentration, but a significant reduction was observed at the IC_60_ concentration.

### 3.7. Immunofluorescence Staining of the BCL-2 L1 and Caspase 3 Proteins

**Figure 8:**
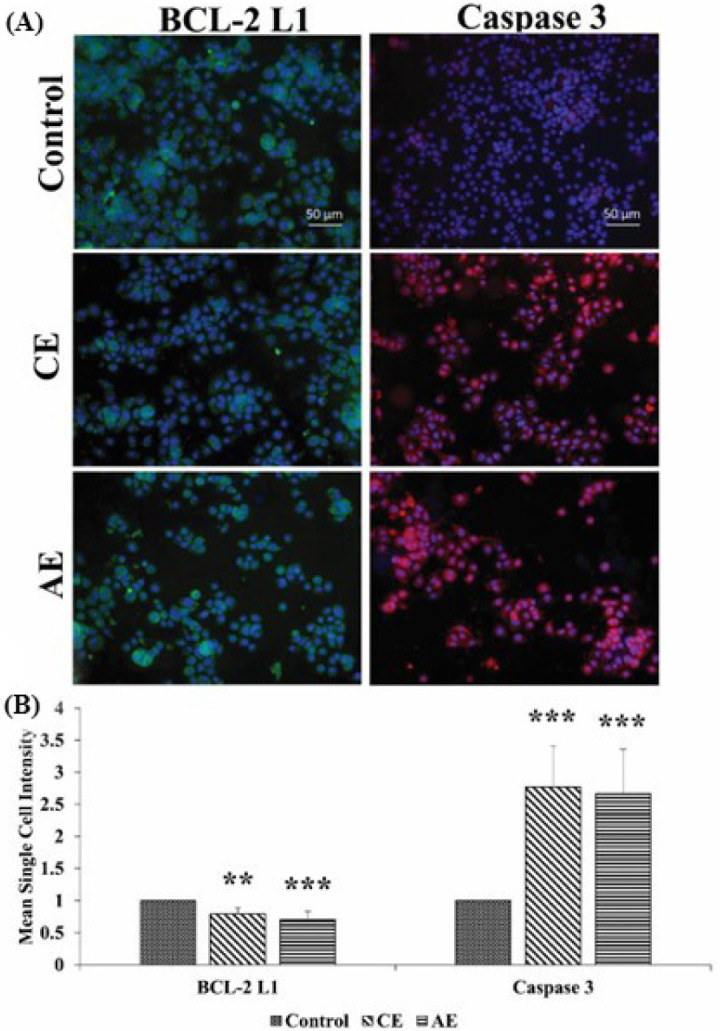
Difference in the expression of the BCL-2 L1 and caspase 3 proteins after the treatment with the compounds (**A**). Mean single cell intensities of each protein at different doses of the compounds (**B**). Each value is the mean ± SD of three experiments. Where the statistically significant *p*-value was considered as: *** *p* < 0.001 and ** *p* < 0.01, as compared with the control. Scale bar: 50 μm.

The monoclonal antibodies against BCL-2 L1 and caspase 3 were used along with fluorochrome attached secondary antibodies, to identify the difference in the protein expressions among the untreated control and the IC_60_ doses of the test compound treated CAL 27 cells (Figure 8). The calculated mean single cell intensities showed a significant decrease in the anti-apoptotic BCL-2 L1 protein and a significant increase in the pro-apoptotic caspase 3 protein in both treatments, compared to the untreated control cells.

### 3.8. Migratory Tendency of the CAL 27 Cells

**Figure 9:**
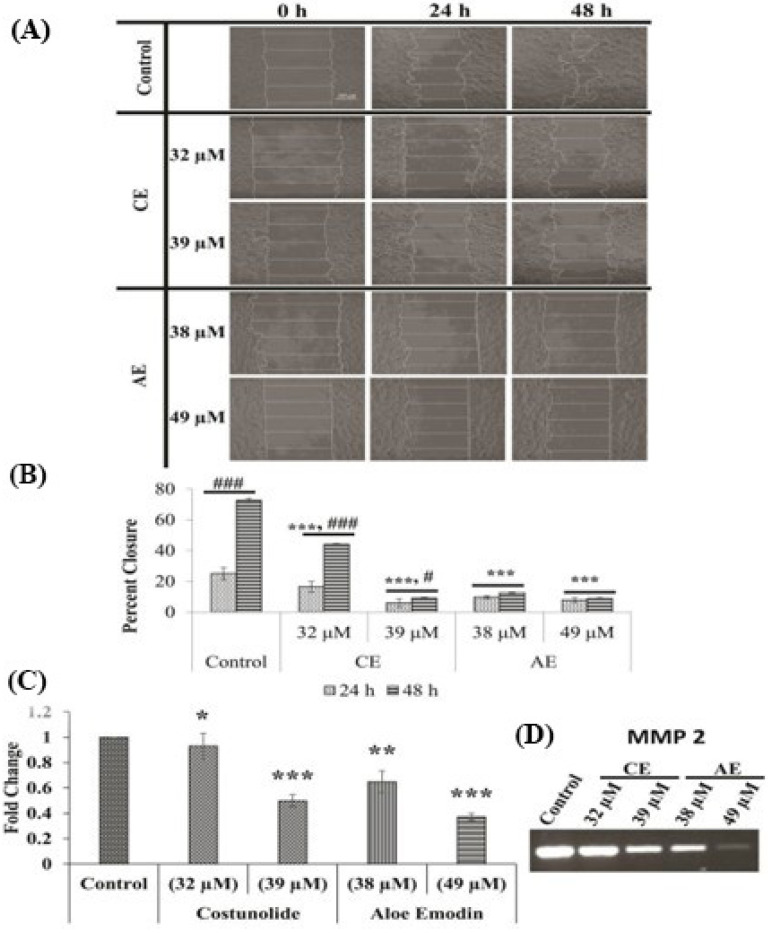
Effect of CE and AE on the migration ability of the CAL 27 cells. The images (**A**) were captured using phase contrast microscopes and the closure percentage of the scratches were calculated and graphically represented (**B**). The gene expression analysis of MMP-2 is shown (**C**,**D**). Each value is the mean ± SD of three experiments. Where the statistically significant *p*-values were considered as: *** *p* < 0.001, ** *p* < 0.01 and * *p* < 0.05, in comparison with the control and ### *p* < 0.001, # *p* < 0.05, in comparison between 24 and 48 h of treatment. Scale bar: 200 μm. The short line above the bars shows same significance. The short line above the bars shows same significance. If both the bars have same significance, we add a short line and put the symbol once.

The untreated CAL 27 cells showed a rapid closure of the scratch up to 25 and 72.6% at 24 and 48 h, respectively. The test compounds reduced the migratory tendency, as the scratches showed a significantly smaller closure at the IC_50_ doses of up to 16.4 and 44%, in the CE treatment, and 9.7 and 12.5%, in the AE treatment, at 24 and 48 h, respectively. A further significant reduction of the closure occurred as the IC_60_ doses of the test compounds increased up to 5.9 and 9%, in the CE treatment, and 7.7 and 8.8%, in the AE treatment, at 24 and 48 h, respectively (Figure 9A,B). Furthermore, both compounds significantly reduced the expression of the pro-metastatic MMP-2 gene, which further compliments the anti-metastatic activity of the test compounds (Figure 9C,D).

## 4. Discussion

Oral cancer is one of the detrimental cancers in Asian countries and accounts for one out of six mortalities worldwide [38]. The side effects related to the available regime of chemotherapy and the emerging resistance have further amplified the complications of this disease, which demands alternative measures to counter oral cancers [39]. Nature has provided us with a diverse series of natural compounds as secondary metabolites with various pharmacological properties, including inhibiting cancer cells. These biocompatible compounds are selective in nature with less toxicity and fewer side effects, which makes them a suitable drug candidate [40]. Two natural compounds (CE and AE) were isolated from the stem of *Lycium shawii* (Figure 1). These compounds were previously reported for diverse biological activities. During the past decade, several studies have reported on the anticancer potential of CE and AE [20,21,22,23,24,25,26]. In this study, AE and CE were evaluated for their anticancer potential against the OSCC cell line (i.e., CAL 27). A colorimetric MTT assay was used to determine the cytotoxicity of AE and CE against the CAL 27 cells and normal mouse fibroblast cells (NIH 3T3) (Figure 2). The IC_50_ of 5-fluorouracil against CAL 27 was 98 µM, however, both AE and CE were found to be active at significantly lower IC_50_ values, i.e., 38 and 32 µM, respectively. Moreover, CE was found to be more potent than AE, in terms of the dose required. AE and CE were found to be less toxic against normal cells with IC_50_ values of >60 µM (Table 1). Further studies to confirm the apoptotic potential and their related mechanisms, were conducted at IC_50_ and IC_60_ (i.e., 32 and 39 μM, respectively, for CE, and 38 and 49 μM, respectively, for AE) which is far less toxic on normal cells.

Apoptosis is the natural phenomenon of cell death which is usually evaded in cancer cells. The process is initiated with the shrinkage of the cell and nucleus, followed by the condensation of the chromatin material (karyopyknosis) and nuclear fragmentation (karyorrhexis). Subsequently, the cell disengages from the surface and loses its specific morphology [41]. A microscopic analysis showed that compounds AE and CE induced the morphological changes in the CAL 27 cells. The phase contrast micrographs showed the treated cells as circular, shrunken, and suspended in the medium, compared with the untreated control cells which retained their morphology (Figure 3). The results are comparable with the previous findings where CE induced the same morphological changes in T24 bladder cancer cells [42]. The morphological changes were further correlated with the nuclear morphological changes, by using a fluorescent nuclear stain, i.e., DAPI. The increased karyopyknosis was observed in the CAL-27 cells, when treated with CE and AE, at IC_50_ and IC_60_ doses (Figure 4). However, karyorrhexis was only observed in the AE treated cells (Figure 4B). A study also showed the presence of karyopyknosis in human lung cancer cells A549, when treated with CE [43]. All of the cellular and nuclear morphological changes, i.e., karyopyknosis, shrinkage, and fragmentation of the cells induced after treatment with AE and CE, are the hallmarks of apoptosis [44]. 

Apoptosis in the treated cells were further validated by a FACS analysis. The analysis was carried out using two very sensitive apoptotic markers: YO-PRO-1 and PI. YO-PRO-1 gives a green fluorescence by selectively passing through the plasma membranes of apoptotic cells, whereas PI labels the necrotic cells with a red fluorescence. The viable cells, moreover, are impermeable for both dyes [45]. This phenomenon can be used to differentiate, not only between the live and dead cells, but also between the necrotic cells and the cells under various stages of apoptosis. The analysis showed significantly higher numbers of the CAL 27 cells in the early and late apoptotic phases, when treated with AE (Figure 5) and CE (Figure 6) at IC_50_ and IC_60_ concentrations. Especially, the AE treated cells showed a greater number of late apoptotic cells at both IC_50_ and IC_60_ doses, i.e., 65.8 and 79.8%, respectively, compared with CE, i.e., 25.06 and 27.51%, respectively. Furthermore, the percentage values of the necrotic cells were very low in the CE test groups, i.e., 2.16 and 4.40%, but in the AE groups, the values are relatively higher, i.e., 25.35 and 14.99% at IC_50_ and IC_60_ doses, respectively. The data strongly emphasize that the test compound’s major cytotoxic effect on the CAL 27 cells was exerted significantly by inducing apoptosis, then necrosis.

During carcinogenesis, various gene or protein expressions are altered in the cellular process, to attain an immortal status attributed by the continuous proliferation, avoiding apoptosis and migration to distant organs [46]. Therefore, in this study, after considering the FACs results, the expression level of various pro-apoptotic (i.e., BAK, caspases 3, and 9) and the anti-apoptotic gene (Bcl-2L1) in the AE and CE treated CAL 27 cells, were deciphered to figure out the possible molecular mechanism of apoptosis (Figure 7). Among the genes, the caspases are the primary mediators of apoptosis, subdivided into different categories, i.e., initiator caspases (caspase 2, caspase 8, caspase 9, and caspase 10), and effector caspases (caspase 3, caspase 6, and caspase 7). They are synthesized as inactive zymogens and are activated upon the intrinsic or extrinsic death signals [47]. Following activation, they alter the mitochondrial membrane integrity, thus, release cytochrome c to induce apoptosis by activating the downstream effector caspases [48]. The anti-apoptotic gen, BCL-2 impedes apoptosis by interacting with the pro-apoptotic Bax and BAK proteins. Upon the death signals, the cytoplasmic level of another pro-apoptotic protein, i.e., BAD, increases, which binds to BCL-2 and releases Bax and BAK, to induce apoptosis ([49]. In the CE treated cells, at both doses, the significantly upregulated expression of the pro-apoptotic genes, i.e., BAK, caspase 6, and 9 was observed. However, the upregulation is higher in the IC_60_ doses, as compared to the IC_50_ doses. Similarly, at the IC_60_ doses, the compound significantly downregulated the expression of the anti-apoptotic gene Bcl-2 L1, but failed to downregulate the gene expression at the IC_50_ level. Moreover, the AE treated cells at both doses, significantly upregulated the pro-apoptotic gene (i.e., BAK, caspases 3, and 6) expression. The expression of the anti-apoptotic gene, i.e., BCL-2 L1, was also significantly downregulated at the IC_60_ concentration. A gene expression analysis was further confirmed by the protein expression analysis of BCL-2 L1 and caspase 3, using immunocytochemistry. The results showed a significant decrease in the BCL-2 L1 protein levels and an increase in the caspase 3 protein levels in the cells treated with IC_60_ doses of both compounds (Figure 8).

Metastasis is another hallmark for the spread of cancer to distant organs, which significantly increases the death probability among patients. Most of the cases are usually diagnosed in the late stages, where the risk of metastasis is relatively high, and the survival rate is only 50% [50,51]. In this regard, CE and AE were also studied for their effect on the cellular motility, by a scratch assay. The results showed a significant reduction in closure percentage of the treated cells, compared with the untreated control cells (Figure 9A,B). Other groups similarly reported the reduced migration by the AE and CE compounds in the different cell lines [52,53]. The metastatic predisposition of the cancer cells is acquired by expressing a family of endopeptidases, i.e., matrix metalloprotease (MMP), which are responsible for the extracellular matrix degradation [54]. Both compounds significantly reduced the expression of MMP-2, at all treatment doses (Figure 9C,D). Another pro-inflammatory gene COX-2 is an important prognostic biomarker of oral cancer and is linked with tumor grading, lymph node metastasis, recurrence, and survival [55]. COX-2 stimulates the cancer cells to secrete angiogenic factors, including FGF-2 and VEGF-A [56]. It favors metastasis by upregulating the expression of MMP-2 and MMP-9 [57]. Reports suggested that the overexpression of the COX-2 gene in OSCC, is associated with a poor prognosis and a decline in the overall 5-year survival rate [58]. The expression of the COX-2 gene was significantly reduced in the treated cells with both test compounds, which suggests the strong anti-metastatic potential of both compounds (Figure 7).

## 5. Conclusions

Conclusively, the study showed that CE and AE had a pronounced apoptotic and anti-migratory potential against the OSCC cells. However, further studies are required to elucidate their complete mechanism of action.

## Figures and Tables

**Table 1:** Half maximal inhibitory concentration of the compounds.

Compounds	IC_50_ ± SD (µM)	
	CAL 27	NIH 3T3
5-flurouracil	97.76 ± 4.5	56.76 ± 2.06
CE	31.99 ± 2.41	61.85 ± 1.91
AE	38.00 ± 2.65	61.13 ± 1.87

## Data Availability

The data presented in this study are available in this manuscript.
